# Effect of Three Apple Rootstocks on the Population of the Small Red-Belted Clearwing Borer, *Synanthedon myopaeformis*

**DOI:** 10.1673/031.006.4001

**Published:** 2006-11-16

**Authors:** Mazen A. Ateyyat

**Affiliations:** Department of Agricultural Sciences, Ash-Shoubak University College, Al-Balqa' Applied University, Al-Salt 19117, Jordan

**Keywords:** burr knot, apple rootstocks M9, M26, MM106

## Abstract

Experiments were conducted in Ash-Shoubak area of Jordan from June 2003 to September 2005 to study the effect of three apple rootstocks on the development of the small red-belted clearwing borer, *Synanthedon myopaeformis* (Borkh.) (Lepidoptera: Sesiidae), under field conditions. Mondial Gala apple trees grafted on the dwarfing rootstock M9 and the semi-dwarfing rootstock M26 were equally infested, whereas those grafted on MM106 showed significantly lower infestation levels. *S. myopaeformis* bore into burr knots that develop below a graft union on rootstocks and girdle the tree. There was a significant effect of rootstock on the numbers of burrs present, and the percentage of burr knots infested by *S. myopaeformis,* with M106 having significantly fewer burrs, and a lower percent infested.

## Introduction

The small red-belted clearwing borer, *Synanthedon myopaeformis* (Borkh.) (Lepidoptera: Sesiidae), is a recent addition to the list of indirect pests of apple in Jordan (Ateyyat 2005). In Europe this insect pest was regarded as one of the secondary pests of apple trees weakened by other factors until the 1960s. It has since become a significant pest and this can be attributed to changes in apple production technology (Balazs et al. 1996). It has become an important insect pest on apple orchards in Jordan (Al-Antary et al. 2005). *S. myopaeformis* has been called the most common sesiid in Jordan and Middle East countries (Ateyyat 2005). It was recorded to attack apple trees in Italy (Balazs et al. 1996), Germany (Dickler 1976), Ukraine (Tertyshny 1995) and Egypt (Abd Elkader and Zaklama 1971). The dogwood borer, *Synanthedon scitula,* and *S. myopaeformis,* are both clear-wing moths in the *Sesiidae* family. Both species bore into burr knots and use these burr knots as oviposition sites (Bergh and Leskey 2003; Riedl et al. 1985; Warner and Hay 1985; Kain and Straub 2001). Burr knots are the result of many partially developed initials that form just below the graft union on some dwarfing and semi-dwarfing rootstocks (Rom 1970; 1973). The larvae of *S. myopaeformis* commonly attack trunks of apple trees on size-controlled rootstocks where burr knots have formed (Dickler 1976). Generally, sessid borers cause a slow decline and reduced yields over several years of infestation due to girdling resulting from feeding in the cambiam layer (Weires 1986).

The main purpose of this research was to study the effect of three apple rootstocks on the development of *S. myopaeformis* under field conditions in Ash-Shoubak in Jordan.

## Materials and Methods

The experiments were conducted in Al-Hashlamoun apple orchards (about 120,000 apple trees) from June 2003 to September 2005 in Ash-Shoubak area (about 1300 m above sea level and 220 km south of Amman) on 10-year old apple trees, *Malus domestica cv.* Mondial Gala grafted on M26, MM106 and M9. Orchardists were requested not to interfere by pesticide application.

### Variations in the populations of *S. myopaeformis*

The numbers of eggs, larvae and pupae were recorded weekly on 12 randomly chosen trees for each rootstock from June 2003 to May 2004. Different trees were sampled each time. Field lenses were used for counting eggs.

### Percentage of infested burr knots

Injury was evaluated on 4 June, 8 August and 15 September 2005 by counting the numbers of infested and non-infested burr knots for each rootstock in one meter of trunk above the soil surface. Four plots of the apple orchards were used for conducting this experiment. Ten trees were randomly chosen for each rootstock in each plot. These 10 trees were considered as one replicate. Infestation was determined by the presence of frass, larva, pupa and/or pupal cases.

### Developmental durations of eggs and pupae under field conditions

According to Al-Antary and Ateyyat (unpublished), *S. myopaeformis* starts oviposition in May and pupation starts in April. Trees were monitored daily during May 2005. Five trees, on which eggs were laid of each rootstock, were monitored until eggs hatched. Due to the daily inspection, the incubation periods were recorded only on 4 of 10 eggs, 4 of 8 eggs, and 5 of 10 eggs laid on MM106, M9 and M26, respectively. To determine the pupation period, over wintered larvae were monitored daily in April 2005. Ten trees on which pupation was seen of each rootstock were monitored until adult emergence.

### Data analysis

Data of variation in populations of *S. myopaeformis* during the experiment were subjected to type I generalized linear model analysis. The percentage of infested burr knots was transformed using arcsine transformation (sin-1x), and subjected to analysis. Analysis was done using SAS 2004 after testing for goodness of fit. Mean separation was calculated, using least significant differences (LSD). The 2D 4v program (Jandel Scientific, 1992) was used to find the linear equation, which describes the relationship between burr knot formation and % infestation.

## Results and Discussion

This is the first report on the effect of apple rootstocks on infestation by eggs, larvae and pupae of *S. myopaeformis*, and the effect of three rootstocks that have different dwarfing characters on number of burr knots and percent infestation of burr knots. The rootstocks used were M9 (dwarfing), M26 (semi-dwarfing) and MM106 (semi-vigorous). *S. myopaeformi* was shown to infest burr knots on the rootstock of trees planted in high-density orchards in Europe (Dickler 1976; Maini and Pasqualini 1980). It is not known what causes burr knot, although a correlation with woolly apple aphid feeding injury has been seen in some apple trees in Ash-Shouback (personal observations). Also, infestation of burr knots by *S. myopaeformis* predisposes the tree to secondary pests that can girdle branches, or even the trunk, weakening the tree. Such a tree is not likely to be productive and should be removed. The infestation of apple trees in eastern North America by the dogwood borer, *S. scitula* (Harris), has been described (Bergh and Leskey 2003; Warner and Hay 1985).

### Percentage of infested burr knots

Mean levels of burr knot infestation in the M9, M26 and MM106 for the early, and mid season are presented in Table 1. Statistically, no significant differences in the percent burr knot infestation were found between the two size-controlling rootstocks; M9 and M26, but both of them had significantly higher infestation than MM106. Both M9 and M26 also showed significantly higher numbers of burr knots compared with numbers of burr knots recorded in MM106 (Table 1). The linear equation that describes the relationship between the number of burr knots and the percent infested burr knots is Y = 0.7X+7.8 (r^2^ = 0.52). Thus, the r^2^ value explains about half of the variation seen in this relationship.

There appears to be an increase in the incidence of burr knots over time as the number of burr knots per tree for M9 in June averaged about 4; in August 6; in September 8 (Table 1). In late-season, M26 showed significantly higher percent infested burr knots compared with MM106 and M9.

**Table 1.**
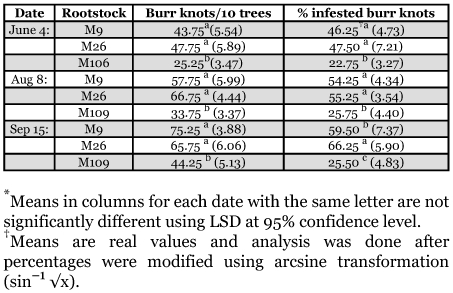
Mean (and standard error) number of burr knots and % infested burr knots with *Synanthedon myopaeformis* under field conditions on three different rootstocks of apple in Ash-Shoubak region in 2005.

These results agree with Leskey and Bergh (2005) in their study on the factors promoting infestation of newly planted apple orchards by the dogwood borer in West Virginia and Virginia. They found that the amount of burr knots have a major effect on the population of this insect. Also, Gut et al. 2005 reported a highly significant association between the rootstock and dogwood infestation. On the other hand, Riedl et al. 1985 reported no association between the number of burr knots with degree of infestation by the dogwood borer. They found that apple trees grafted on MM111 showed significantly lower infestation levels with dogwood borer than those trees grafted on M9, M26, MM106 that were equally infested with dogwood borer. However, Gut et al. 2005 discussed the differences between their results and those of Riedl et al. 1985, in that the latter researchers recorded the infestation on the same rootstock, but with different scions.

### Developmental duration of eggs and pupae

The duration of the egg period during May of 2005 varied from 7–9 days on the three rootstocks (Table 2). The average daily temperature and relative humidity during May 2005 were 16°C and 26%, respectively. The developmental periods of pupae during April varied between 30.2 to 33.7 days on the three rootstocks (Table 2). The average daily temperature and relative humidity during April 2005 were 13°C and 43%, respectively.

**Figure 1.**
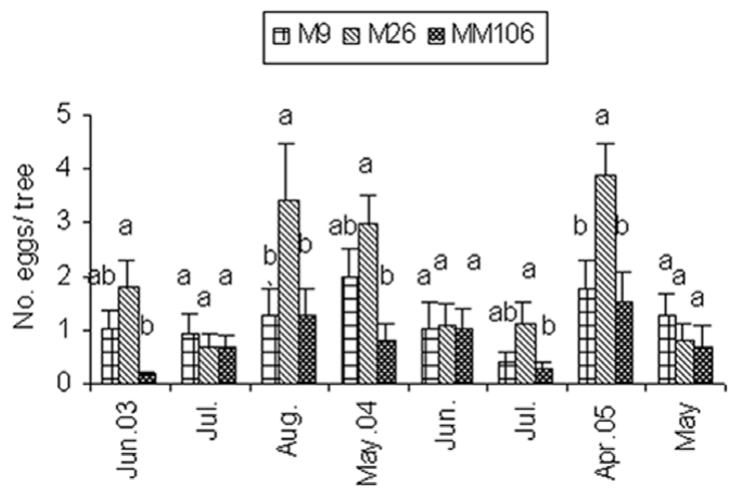
Monthly mean numbers of eggs of Synanthedon myopaeformis per tree (±SE) on Mondial Gala apple branches grafted on MM 106, M26 and M9 rootstocks in the Ash-Shoubak area from June 2003 to May 2005.

**Table 2.**
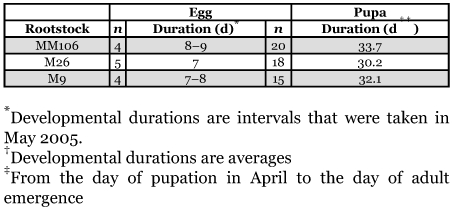
Developmental durations for eggs and pupae of *Synanthedon myopaeformis* under field conditions on three different rootstocks of apple in Ash-Shoubak region in 2005

### Variation in populations of *S. myopaeformis*

No eggs were recorded from September to April during the study period (Figure 1). No significant differences in the number of eggs found in the three studied rootstocks were recorded in July 2003, June 2004 and May 2005 but significantly high numbers of eggs were recorded on M26 in August 2003 and April 2005 (Figure 1). M9 showed significantly higher numbers of larvae on June 2003, and M26 showed significantly higher numbers of larvae in August 2003, and from October 2004 to January 2005 (Table 3). No other significant differences were seen.

The above reported results show that there was a significant effect of apple rootstock on the number of burr knots present and the percentage of burr knot infestation by *S. myopaeformis.* Since 1990s, *S. myopaeformis* has been recognized as secondary pest of apple in Ash-Shoubak region that represents the most important apple planting area of Jordan (Al-Antary et al. 2005). But its importance was increased due to the increased popularity of commercial dwarfing and semi-dwarfing clonal rootstocks. Several control measures have been proposed for controlling sessid borers. Ateyyat (2005) suggested using Chlormezyl® or Actellic® for controlling *S. myopaeformis* larvae in Jordan.

**Table 3.**
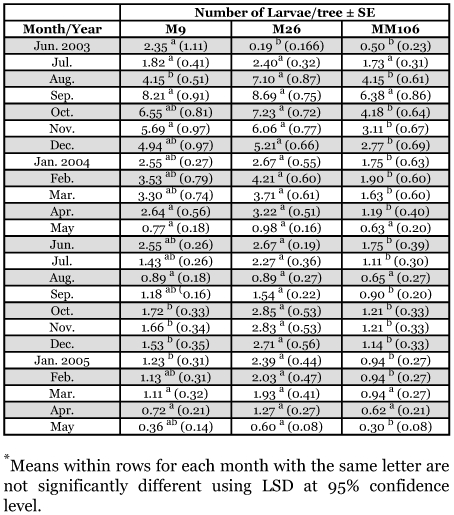
Monthly mean numbers of larvae of *Synanthedon myopaeformis* per tree (and standard error) on Mondial Gala apple grafted on MM 106, M26 and M9 in Ash-Shoubak area from June 2003 to May 2005.

**Table 4.**
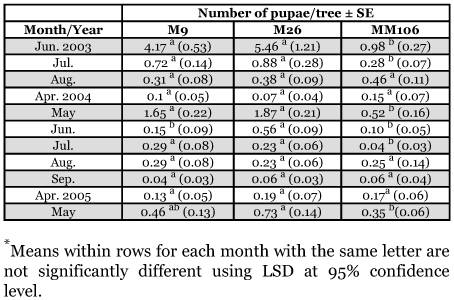
Monthly mean numbers of pupae of *Synanthedon myopaeformis* per tree (and standard error) on Mondial Gala apple grafted on MM 106, M26 and M9 in Ash-Shoubak area from June 2003 to May 2005

Gut et al. (2005) showed that mounding of soil to cover the exposed rootstock was found to be a highly effective alternative to insecticides for dogwood borer control. But, Leskey and Bergh (2005) found that the infestation of the rooted tissue returned during the same season after the removal of the mounds. It is difficult to prevent farmers from planting apple cultivars on dwarfing and semi-dwarfing clonal rootstocks. Even though pesticides may provide satisfactory control of this insect, but they have negative effects on humans and cause pollution to the environment if used improperly. Testing other control measures, such as the use of pheromone traps, is appropriate.
